# Association of co-exposure to extreme cold and nitrogen dioxide with psoriasis outpatient visits and systemic inflammation

**DOI:** 10.3389/fpubh.2026.1916342

**Published:** 2026-09-09

**Authors:** Yuting Huang, Suo Mo, Bokun Zhu, Haili Ren, Yajuan Zuo, Yuxiao Zhou, Min Zhang, Xuemei Zhou, Jiemiao Shen, Minghui Ji, Xianfeng Cheng

**Affiliations:** 1 School of Nursing, Nanjing Medical University, Nanjing, China; 2 Department of Laboratory, Hospital for Skin Diseases, Institute of Dermatology, Chinese Academy of Medical Sciences & Peking Union Medical College, Nanjing, China; 3 Department of Network Information Center, Hospital for Skin Diseases, Institute of Dermatology, Chinese Academy of Medical Sciences & Peking Union Medical College, Nanjing, China; 4 The Fourth Clinical College, Nanjing Medical University, Nanjing, China

**Keywords:** co-exposure, extreme cold, nitrogen dioxide, psoriasis, systemic inflammation

## Abstract

**Background:**

Psoriasis, a chronic inflammatory skin disorder, is closely linked to environmental triggers. However, the combined effects of extreme cold and nitrogen dioxide (NO_2_), two common environmental factors, on psoriasis patients remain unclear. This study aims to explore the associations of co-exposures to extreme cold and NO_2_ with psoriasis outpatient visits and systemic inflammatory markers among patients with complete blood count (CBC).

**Methods:**

Data from patients with psoriasis were collected in a subtropical megacity of China between January 2021 and December 2025. A distributed lag non-linear model (DLNM) was used to evaluate the outpatient visit risk associated with extreme cold, extreme NO_2_, and their co-exposure, identifying susceptible subgroups. Additive interaction was assessed using an interaction term and the relative excess risk due to interaction (RERI). Furthermore, to evaluate the effect of co-exposure on systemic inflammatory markers, a sub-cohort of patients with CBC records was extracted for exploratory analysis.

**Results:**

A total of 63,411 psoriasis outpatient visits and 3,048 patients who underwent CBC tests were recorded during the cold seasons of 2021–2025. Co-exposure to extreme cold and NO_2_ was associated with an increased risk of psoriasis outpatient visits at Lag14 (RR = 1.017, 95% CI: 1.004–1.030), with a higher point estimate compared to single-factor exposure; however, the interaction test was not significant (RERI = 0.026, 95% CI: −0.055 to 0.108). Stratified analysis revealed that the association between co-exposure and outpatient visits varied by age, with a stronger effect observed in younger age groups (Q-test for interaction, *p* = 0.004). In exploratory analyses, SII showed a significant global association (*p* = 0.016) at Lag7 (OR = 1.109, 95% CI: 1.031–1.194), whereas NLR and PLR were not significant.

**Conclusion:**

Co**-**exposure to extreme cold and NO_2_ is associated with an increased risk of psoriasis outpatient visits, particularly in susceptible age groups, although no synergistic effect was detected between the two exposures. Exploratory analyses also suggest a potential association with elevated systemic inflammation, warranting further investigation. These findings provide preliminary evidence for the role of combined environmental exposures in psoriasis and highlight the importance of considering age-specific vulnerability when developing targeted public health interventions for at-risk populations.

## 1 Introduction

Psoriasis is a globally prevalent chronic immune-mediated inflammatory disease, affecting approximately 125 million psoriasis patients worldwide (1). It is characterized by abnormal keratinocyte proliferation, inflammatory cell infiltration, relapsing–remitting cutaneous lesions, and persistent systemic inflammation, which not only disrupt skin homeostasis but also elevate the risk of comorbidities including cardiovascular disease and metabolic syndrome (2). According to Global Burden of Disease (GBD) study 2021, the number of prevalent cases of psoriasis in China has reached 8.45 million (3). Given that psoriasis is incurable and highly prone to relapse, patients require an average of 8.7 outpatient visits per year (4). This not only seriously damages physical and mental health but also causes a massive economic and medical burden (5). Therefore, identifying and controlling the risk factors that exacerbate psoriasis is crucial for mitigating the overall disease burden.

Psoriasis exacerbation is influenced by multiple factors, including genetic predisposition, immune dysfunction, and environmental triggers (6, 7), among which environmental factors have attracted increasing attention due to their modifiability. Air pollution and meteorological factors are important preventable risk factors for the onset and development of psoriasis (8). Nitrogen dioxide (NO_2_), a typical gaseous pollutant primarily emitted from vehicle exhaust, industrial processes, and fossil fuel combustion, is an important precursor for ozone (O_3_) formation and secondary particulate matter (PM_2.5_) generation (9). It can penetrate the respiratory tract and induce systemic oxidative stress and inflammation (10). Epidemiological research confirms that short-term exposure to NO_2_ can significantly increase outpatient visits for psoriasis (11–13). Extreme cold events, another common environmental stressor, have been linked to inflammatory responses that exacerbate psoriasis symptoms. Cold stress disrupts skin barrier function, activates immune cells, and promotes the release of pro-inflammatory cytokines (14). This aligns with the disease’s pronounced seasonal pattern which is characterized by exacerbation in winter and remission in summer (15) because low temperatures further induce pro-inflammatory cell production and inhibit skin barrier repair (14, 16). In regions such as Nanjing, China, extreme cold occurs frequently in winter, and this period often coexists with elevated NO_2_ concentrations due to increased residential heating, fossil fuel combustion, and industrial loads (17). Inversion layers further hinder NO_2_ diffusion (18). However, while previous studies have shown that co-exposure to extreme heat and air pollutants significantly increases mortality risks and disease burdens (19, 20), the combined effects of extreme cold and NO_2_ on psoriasis patients remain largely unexplored.

Systemic inflammation is widely recognized as a crucial indicator of psoriasis severity, with indices such as the neutrophil-to-lymphocyte ratio (NLR), platelet-to-lymphocyte ratio (PLR), and systemic immune-inflammation index (SII) closely associated with the psoriasis area and severity index (PASI) (21–23). The general psoriasis outpatient population reflects the overall burden of disease exacerbation caused by environmental factors, while moderate-to-severe psoriasis patients are more prone to severe systemic inflammation, which is a key feature of disease severity and comorbidity risk (22, 24). They are also the group where hematological abnormalities predominate, especially during acute exacerbations requiring systemic intervention (25). Previous studies have shown that both NO_2_ exposure and cold stimulation can induce oxidative stress and elevate systemic inflammatory markers (26–28). However, most existing research has focused on the effects of environmental exposure on outpatient visits, with limited investigation into how such exposure drives disease progression from an inflammatory perspective. Consequently, it remains unclear whether co-exposure to extreme cold and NO_2_ triggers immune imbalances and inflammatory responses in vulnerable patients.

Accordingly, based on psoriasis outpatient records in a subtropical megacity, this study aims to investigate and compare the delayed effects of extreme cold, extreme NO_2_, and their co-exposure on outpatient visits, as well as to identify susceptible subgroups. Furthermore, we extracted data from outpatients’ complete blood count (CBC) test records to exploratorily assess the effect of the co-exposure on systemic inflammatory indices, such as NLR, PLR, and SII. This study will provide a comprehensive scientific foundation for seasonal protection and early clinical interventions for psoriasis.

## 2 Materials and methods

### 2.1 Study location and population

Nanjing, one of the subtropical megacities of China, is the capital of Jiangsu Province (31°14′-32°37′N, 118°22′-119°14′E), with a total area of 6,587.04 km^2^ and a permanent population of over 9.3 million. The city experiences a northern subtropical monsoon climate, characterized by cold, dry winters and hot, humid summers, with an annual average temperature of 15.4 °C (extremes ranging from −13.1 °C to 39.7 °C) (20). As a megacity, Nanjing serves as a national transportation hub and a major industrial base in East China, where traffic and industrial emissions constitute the primary sources of air pollution (29).

Psoriasis outpatient data in Nanjing from January 1, 2021, to December 31, 2025, were collected from the electronic medical record system of the Hospital for Skin Diseases, Chinese Academy of Medical Sciences and Peking Union Medical College. All outpatient diagnoses of skin diseases were made by professional dermatologists at this hospital. Patients diagnosed with psoriasis were included based on the International Classification of Diseases, 10th Revision (ICD-10) code L40. We excluded patients with unclear diagnoses, incomplete information, primary diagnoses other than psoriasis, or those who were not permanent residents of Nanjing. Each data entry represents a single visit record for a patient, from which clinical characteristics including sex, age, and date of visit were collected. To protect patient privacy, this study did not involve personal information such as names, identification numbers, or contact details. This study complied with the principles of the Declaration of Helsinki, and the requirement for informed consent was waived. Furthermore, a subgroup of the outpatient population who underwent CBC tests for systemic inflammatory markers was included for exploratory analysis. Patients with acute infections, autoimmune diseases other than psoriasis, or severe organ dysfunction were excluded from this subgroup to avoid confounding.

### 2.2 Meteorological and air pollution data

Daily meteorological and air quality records from January 1, 2021, to December 31, 2025, were integrated into this study. Daily mean temperature (°C) and relative humidity (%) were collected from the China Meteorological Data Service Centre.^1^ Air pollutant data were obtained from the National Urban Air Quality Real-time Publishing Platform.^2^ These data included daily 24-h average concentrations of particulate matter with an aerodynamic diameter of less than 10 μm (PM_10_), particulate matter with an aerodynamic diameter of 2.5 μm or less (PM_2.5_), nitrogen dioxide (NO_2_), sulfur dioxide (SO_2_), and carbon monoxide (CO), as well as the daily maximum 8-h average concentration of ozone (O_3_).

### 2.3 Relevant definition

#### 2.3.1 Extreme events

Currently, unified definitions for extreme cold and extreme NO_2_ are lacking. Research suggests that individuals can adapt to their local ambient temperatures to a certain extent (30). Consequently, the health impacts of extreme temperatures vary by region and are typically defined relative to local averages rather than absolute values. Similarly, air pollutant extremes are often defined using top percentiles. Therefore, following established methods from previous studies (19), we defined extreme cold using the 10th (T10), 7.5th (T7.5), and 5th (T5) percentiles of daily mean temperature in Nanjing from 2021 to 2025, and defined extreme NO_2_ levels using the 90th (N90), 92.5th (N92.5), and 95th (N95) percentiles of daily 24-h mean concentrations over the same period.

Co-exposure events were identified when extreme cold and elevated NO_2_ occurred on the same day, yielding three co-exposure definitions: T10_N90, T7.5_N92.5, and T5_N95. Specifically, T10_N90 indicates days when daily mean temperature ≤ 10th percentile and NO_2_ daily 24-h mean concentrations ≥ 90th percentile; T7.5_N92.5 represents days with temperature ≤ 7.5th percentile and NO_2_ concentrations ≥ 92.5th percentile; and T5_N95 refers to days with temperature ≤ 5th percentile and NO_2_ concentrations ≥ 95th percentile. T10_N90 was selected as the primary definition for the main analysis, while T7.5_N92.5 and T5_N95 were used to assess the robustness of the findings to alternative exposure thresholds.

Given that both extreme cold and high NO_2_ levels primarily occur in winter, the study period was restricted to the cold season (November to March of the following year).

#### 2.3.2 Definition of high systemic inflammatory

Previous research indicates that indicators such as NLR, PLR, and SII are affected by multiple factors including age, sex, ethnicity, lifestyle and laboratory equipment, which leads to considerable inter-individual variation (31). Consequently, different cutoff values have been reported across various diseases, and even studies on the same disease often show a lack of consistency (32). As there are currently no globally recognized absolute reference ranges for these indicators, we adopted a definition based on statistical distribution, following established methodologies. Specifically, we calculated quartiles based on the distribution of these indicators within our total sample and defined patients in the upper quartile (Q4, > 75th percentile) as having “high systemic inflammation levels.”

### 2.4 Statistical analysis

Spearman correlation analysis was performed to examine the relationships between meteorological factors and air pollutants; a correlation coefficient of |*r*| > 0.7 was considered to indicate a strong association. Distributed lag non-linear models (DLNM) combined with the generalized additive model (GAM) were employed to evaluate the exposure-lagged response associations between extreme cold, NO_2_, co-exposure and daily psoriasis outpatient visits (33, 34). We defined a maximum lag of 21 days to account for delayed effects (35). The lag dimension of the cross-basis was fitted using the natural cubic spline function and had a degree of freedom of 3. To address overdispersion, negative binomial regression was applied. The model fitting was performed using restricted maximum likelihood estimation. The models were adjusted for relative humidity (RH), air pollutants, calendar time, day of the week (DOW), holidays and the date of clinic closure (Hospital_day). The model is specified as follows:


$$\begin{array}{c} \log[E(Y_{t})]=\alpha +\mathrm{cb}(X_{t})+s(RH_{t},k=4)+s(PM_{2.5},k=4)\\ +s(\mathrm{CO},k=4)+s(\text{Time},k=5)+\text{factor}(\mathrm{Do}w_{t})\\ +\text{Holida}y_{t}+\text{Hospital}\_\mathrm{da}y_{t} \end{array}$$

In this model, *E(Y_t_)* represents the expected outpatient volume on day *t*; *α* the intercept; and *cb*(*X_t_*) denotes the cross-basis matrix. The function *s* represents smooth penalized splines, with degrees of freedom (*k*) used to flexibly control for non-linear confounding. Based on correlation screening in the Supplementary Table S1, RH, PM_2.5_, and CO were selected as covariates and included as continuous smooth functions with basis function dimension *k* = 4. Among the traffic-related pollutants, PM_2.5_ and CO were chosen as the primary adjustment variables because they showed the strongest correlations with NO_2_ (*r* = 0.561 and *r* = 0.582, respectively) and are well-established confounders in environmental epidemiological studies (Supplementary Table S1). PM_10_ was excluded from the main model due to its high correlation with PM_2.5_, which would introduce substantial collinearity if both were included simultaneously. A sensitivity analysis substituting PM_2.5_ with PM_10_ was performed to evaluate the robustness of the results. SO_2_ and O_3_ were not included in the primary model but were examined in sensitivity analyses to assess the robustness of our findings to additional adjustments. Calendar time was modeled using a basis function with *k* = 5 to capture the long-term trend. The days of the week (DOW) and public holidays were adjusted as categorical variables, and Hospital_day (a binary indicator for clinic closure) was introduced to control for the bias caused by interruption in visits. Diagnostic tests for collinearity and curvature (VIF < 5 and the worst-case concurvity < 0.8) confirmed that the model did not have serious multicollinearity issues (Supplementary Table S2) (36).

In order to investigate the interaction effect between extreme cold and NO_2_ exposure, we fitted an interaction model. Specifically, we defined T10_N90 as the primary exposure classification, where the four mutually exclusive categories were: 0 = neither exposure (reference group), 1 = only high NO_2_ (N90), 2 = only extreme cold (T10), and 3 = both exposures (T10_N90). To assess the robustness of our findings to alternative exposure definitions, we additionally constructed two sensitivity interaction models using T7.5_N92.5 and T5_N95 as alternative thresholds for extreme cold and NO_2_, respectively. The covariates and smoothing parameters were exactly the same as the main model, as follows:


$$\begin{array}{c} \log[E(Y_{t})]=\alpha +\beta (T\_N_{t})+s(RH_{t},k=4)+s(PM_{2.5},k=4)\\ +s(\mathrm{CO},k=4)+s(\text{Time},k=5)+\text{factor}(\mathrm{Do}w_{t})\\ +\text{Holida}y_{t}+\text{Hospital}\_\mathrm{da}y_{t} \end{array}$$

*T_N_t_* is a four-category variable, *β* represents the corresponding regression coefficient vector. The model was fitted using negative binomial regression to handle the excessive dispersion of daily count data. Based on this model, we separately estimated the relative risk (RR) of only T10 (RR_10_), only N90 (RR_01_), and T10_N90 (RR_11_) compared to the reference group. To evaluate additive interaction, we calculated the relative excess risk due to interaction (RERI) using the following formula:


$$\text{RERI}=RR_{11}-RR_{10}-RR_{01}+1$$

A RERI value greater than 0 indicates a positive additive interaction, suggesting a synergistic effect. The 95% confidence interval of RERI is estimated using the Delta method to determine whether the interaction is statistically significant (19).

Stratified analyses by sex (male, female) and age (<18, 18–45, 46–65, and >65 years) were performed based on the main model to identify potentially susceptible populations (13). The difference between sexes was tested using a *Z*-test, while heterogeneity across the four age groups was assessed using Cochran’s *Q*-test.

To assess the robustness of our results, sensitivity analyses were conducted by: (1) applying 14-day and 28-day washout periods to exclude potential repeat visits. (2) removing all air pollutant covariates from the model, substituting PM_10_ for PM_2.5_ as the particulate matter covariate, including SO_2_ and/or O_3_ as additional covariates in the model. (3) Varying the degrees of freedom for calendar time (*k* = 4–7). Effect estimates at Lag14 were compared against the primary model to evaluate result stability.

Finally, to evaluate the effects of extreme cold, high NO_2_, and their co-exposure on systemic inflammatory markers, we analyzed the CBC data from the psoriasis patients. Systemic inflammation levels, as reflected by NLR, PLR, and SII, were dichotomized using the 75th percentile (Q4) of their respective distributions as the threshold for elevated markers. To examine these associations, a quasi-binomial model in the GAM with a logit link was fitted. This model used the number of patients with daily elevated inflammatory markers as the numerator, the total daily CBC as the denominator, to address the issues of daily detection quantity fluctuations and excessive dispersion. The model is set as follows:


$$\begin{array}{c} \log(\frac{p_{t}}{1-p_{t}})=\alpha +\mathrm{cb}(T10\_N90_{t})+s(RH_{t},k=4)\\ +s(PM_{2.5},k=4)+s(\mathrm{CO},k=4)+s(\text{Time},k=5)\\ +\text{factor}(\mathrm{Do}w_{t})+\text{Holida}y_{t} \end{array}$$

*p_t_* represents the conditional probability of high inflammatory markers on the *t*-th day in the population undergoing CBC tests; *cb*(*T10_N90_t_*) is the cross-basis matrix of co-exposure (with a maximum lag period of 21 days, the lag dimension using natural cubic splines, *df* = 3); the model adopts the logit link function.

Given that psoriasis typically reaches its peak inflammatory response around 1 week following exposure to external triggers (37), Lag7 was selected as the primary observation window for the exploratory analysis of systemic inflammatory markers. For outpatient visits, patients may delay seeking medical attention due to self-monitoring and self-medication. Therefore, Lag14 was selected as the main reporting window. Both lag windows were chosen based on clinical reasoning rather than data-driven selection.

All statistical analyses were conducted using the R software (version 4.5.0) and the “dlnm” package. The analysis code is available in Supplementary Data Sheet 1. The effect estimates in the main outpatient visit analysis were expressed as relative risk (RR) and accompanied by 95% confidence intervals (CI). For the exploratory analysis of systemic inflammatory markers, the effect estimates were expressed as odds ratio (OR) and accompanied by 95% CI.

## 3 Results

### 3.1 Data descriptions

A total of 63,411 psoriasis outpatient visits were recorded during the study period. Male patients accounted for 68.5% (43,440 visits) of the total. Among all age groups, the 18–45 age group had the highest frequency of visits, representing 43.2% (27,375 visits), while the <18 age group had the lowest frequency, accounting for 2.6% (1,679 visits). A total of 3,048 CBC tests were performed for psoriasis patients. Regarding meteorological conditions, the daily mean temperature was 8.5 °C, with a daily mean relative humidity of 65.1%. For air pollutants, the mean daily concentrations of PM_10_, PM_2.5_, NO_2_, SO_2_, O_3_, and CO were 75.2 μg/m^3^, 39.5 μg/m^3^, 37.6 μg/m^3^, 6.4 μg/m^3^, 54.6 μg/m^3^, and 0.7 μg/m^3^, respectively (Table 1).

**Table 1:** Daily outpatient visits for psoriasis, meteorological and air pollutants data (November–March, 2021–2025).

Variables	Count	Mean (SD)	Median	IQR	Range
Daily outpatient visits	63,411	83.9 (44.1)	95	68.3–117	0–168
Visits by sex					
Male	43,440	57.5 (30.7)	65	46–80.8	0–114
Female	19,971	26.4 (14.5)	29	21–37	0–58
Visits by age					
< 18 years	1,679	2.2 (2.2)	2	0–3	0–11
18–45 years	27,375	36.2 (19.8)	40	29–49	0–85
46–65 years	24,414	32.3 (17.8)	36	24–45	0–66
> 65 years	9,943	13.2 (8.7)	14	7–20	0–39
Daily blood cell test	3,048	4 (3.2)	4	1–6	0–17
Meteorology					
Temperature (°C)	–	8.5 (5.3)	8.1	4.7–11.9	−4.8 to 24.3
Relative humidity (%)	–	65.1 (16.6)	64	52–77	22.5–100
Air pollutants (μg/m^3^)					
PM_10_	–	75.2 (37.2)	71	48–95.8	11–405
PM_2.5_	–	39.5 (21.6)	35	23–52	5–123
NO_2_	–	37.6 (17.3)	35	25–48	3–126
SO_2_	–	6.4 (2.6)	6	4–8	1–21
O_3_	–	54.6 (20.9)	53	41–67	3–133
CO	–	0.7 (0.2)	0.7	0.5–0.8	0.1–1.8

SD, standard deviation; IQR, interquartile range; PM_2.5_, particulate matter with an aerodynamic diameter of 2.5 μm or less; PM_10_, particulate matter with an aerodynamic diameter of less than 10 μm; NO_2_, nitrogen dioxide; SO_2_, sulfur dioxide; CO, carbon monoxide; O_3_, ozone.

**Figure 1:**
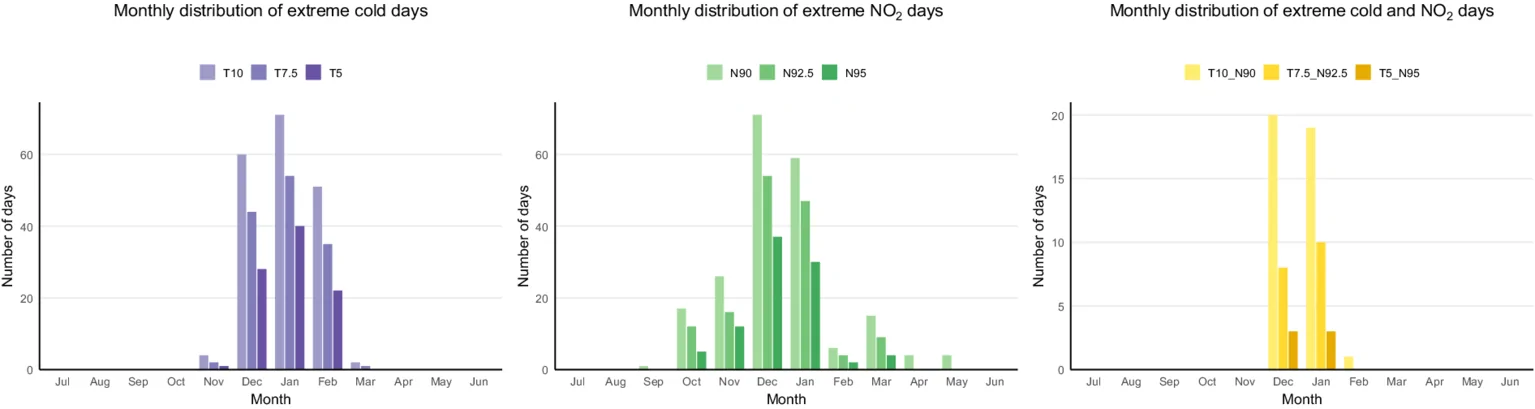
Distribution of extreme cold days, extreme NO_2_ days and co-exposure days at different percentile thresholds by month. Threshold definitions: T10: Daily mean temperature ≤ 10th percentile (6.1 °C); N90: Daily mean NO_2_ ≥ 90th percentile (42 μg/m^3^); T10_N90: Both temperature ≤ 10th percentile and NO_2_ ≥ 90th percentile; T7.5: Daily mean temperature ≤ 7.5th percentile (5.4 °C); N92.5: Daily mean NO_2_ ≥ 92.5th percentile (45 μg/m^3^); T7.5_N92.5: Both temperature ≤ 7.5th percentile and NO_2_ ≥ 92.5th percentile; T5: Daily mean temperature ≤ 5th percentile (4 °C); N95: Daily mean NO_2_ ≥ 95th percentile (50 μg/m^3^); T5_N95: Both temperature ≤ 5th percentile and NO_2_ ≥ 95th percentile.

Extreme cold days, extreme NO_2_ days, and their co-exposure days exhibited distinct seasonal characteristics, primarily concentrated in the winter months from November to March (Figure 1).

### 3.2 Association between extreme events and outpatient visits for psoriasis

Extreme cold and NO_2_ levels were defined using percentile-based thresholds (T10: temperature ≤ 6.1 °C; N90: NO_2_ ≥ 42 μg/m^3^). The detailed cutoff values, number of exposure days, and corresponding outpatient visit situations for all thresholds are presented in Supplementary Table S3, as well as the model diagnostic indicators, including the discrete parameter θ and AIC values, which remained stable across different thresholds.

**Figure 2:**
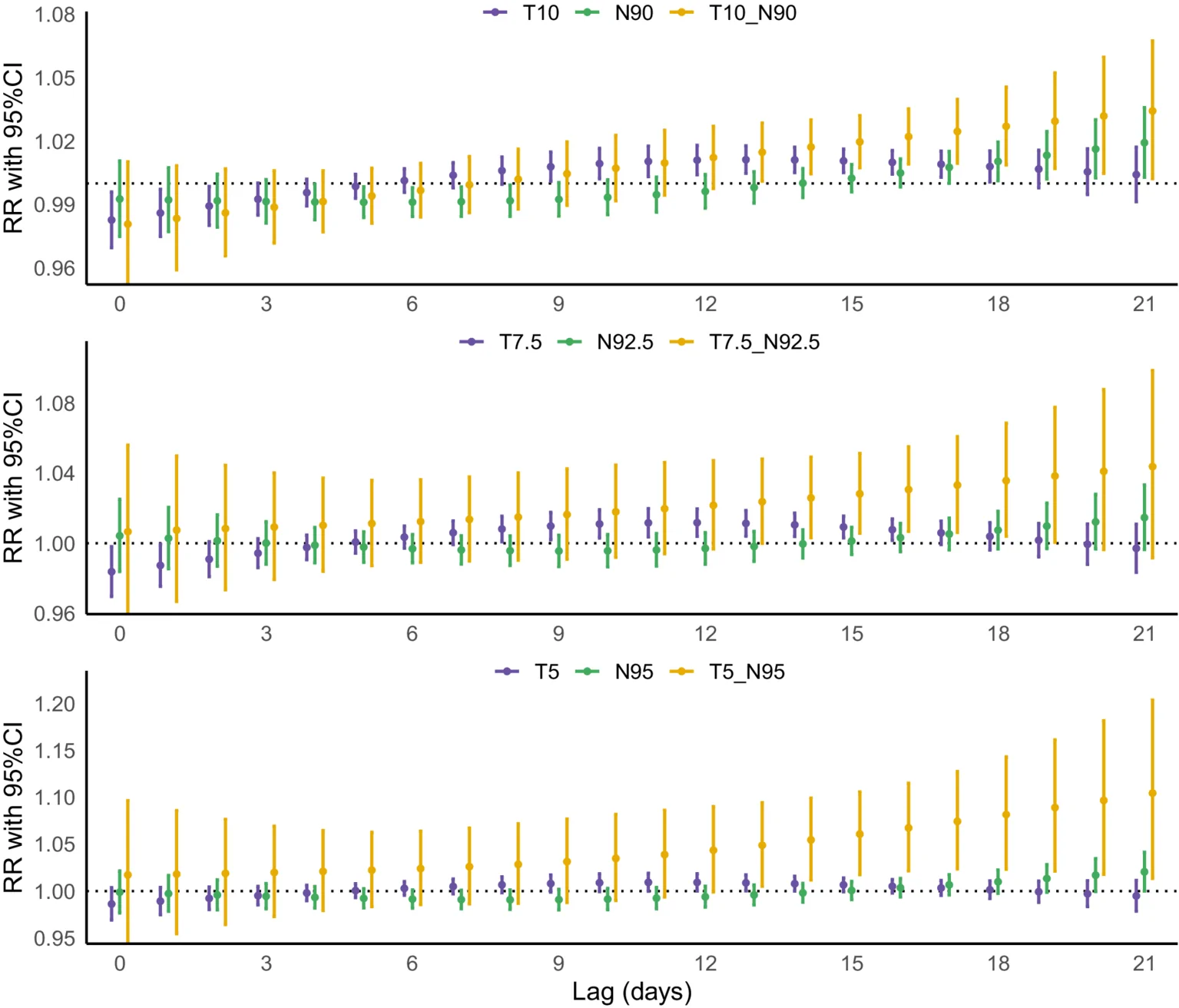
Single-day lag effects of extreme cold, extreme NO_2_ and co-exposure at different percentile thresholds on the outpatient visits for psoriasis. Threshold definitions: T10: Daily mean temperature ≤ 10th percentile (6.1 °C); N90: Daily mean NO_2_ ≥ 90th percentile (42 μg/m^3^); T10_N90: Both temperature ≤ 10th percentile and NO_2_ ≥ 90th percentile; T7.5: Daily mean temperature ≤ 7.5th percentile (5.4 °C); N92.5: Daily mean NO_2_ ≥ 92.5th percentile (45 μg/m^3^); T7.5_N92.5: Both temperature ≤ 7.5th percentile and NO_2_ ≥ 92.5th percentile; T5: Daily mean temperature ≤ 5th percentile (4 °C); N95: Daily mean NO_2_ ≥ 95th percentile (50 μg/m^3^); T5_N95: Both temperature ≤ 5th percentile and NO_2_ ≥ 95th percentile. All estimates were adjusted for RH, PM_2.5_, CO, time trend, DOW, holidays, and hospital closure days.

Compared with non-extreme events, co-exposure to extreme cold and NO_2_ was associated with a significantly increased risk of psoriasis outpatient visits at Lag14 (RR = 1.017, 95% CI: 1.004–1.030; global *p* = 0.008). The single-day and cumulative lag RR of co-exposure was higher than that of single-factor exposure (Figure 2; Supplementary Table S4). However, testing for additive interaction using the RERI revealed no significant synergistic effect at Lag14 (RERI = 0.026, 95% CI: −0.055 to 0.108). Isolated significant positive interactions were observed at Lag6 for T10_N90 and at Lag5 for T5_N95 (Supplementary Table S5).

### 3.3 Effect modification by sex and age

In the sex-stratified analysis, the effect estimate for co-exposure was slightly higher in males (RR = 1.020, 95% CI: 1.007–1.034) than in females (RR = 1.009, 95% CI: 0.993–1.026). However, the formal test of interaction was not statistically significant (*p* = 0.314), indicating no significant effect modification by sex. In the age-stratified analysis, the RR showed a decreasing trend with increasing age. Higher estimates were observed in the < 18 years group (RR = 1.047, 95% CI: 0.992–1.104) and the 18–45 years group (RR = 1.033, 95% CI: 1.017–1.049), whereas the estimates in the 46–65 years group and the > 65 years group were 1.012 (0.997–1.027) and 0.985 (95% CI: 0.964–1.007), respectively. The Cochran’s *Q*-test for heterogeneity across the four age groups was significant (*p* = 0.004) (Table 2; Supplementary Tables S6, S7).

**Table 2:** Relative risks for T10_N90 co-exposure at Lag14, stratified by sex and age group.

Subgroups	Relative risk	95% CI	*P* for interaction
Sex			
Male	1.020	(1.007,1.034)	0.314
Female	1.009	(0.993,1.026)	
Age			
< 18 years	1.047	(0.992,1.104)	0.004
18–45 years	1.033	(1.017,1.049)	
46–65 years	1.012	(0.997,1.027)	
> 65 years	0.985	(0.964,1.007)	

P for interaction was estimated using the *Z*-test for sex (male vs. female) and the *Q*-test for age (across four groups), assessing whether the effect of co-exposure (T10_N90) differed significantly across subgroups at Lag14.

### 3.4 Sensitivity analysis

We conducted a sensitivity analysis to assess the robustness of our findings. These included exclusion of repeated visits within the 14-day and 28-day washout periods (Supplementary Table S8), a single-pollutant model without co-pollutant adjustment, substituting PM_2.5_ with PM_10_, additionally adjusting for SO_2_, O_3_, or both (Supplementary Table S9), and varying the degrees of freedom of the time trend (Supplementary Table S10). The estimated values of the extreme co-exposure effect were largely consistent with those of the main model.

### 3.5 Exploratory analysis of the association between extreme cold and NO_2_ co-exposure and systemic inflammatory markers in psoriasis outpatients

In the exploratory analysis of systemic inflammatory markers among outpatients who underwent CBC testing using the quasi-binomial GAM, the global test results of the cross-basis items were not significant for NLR (*p* = 0.331) and PLR (*p* = 0.834), but reached statistical significance for SII (*p* = 0.016). The complete single-lag period estimates are presented in Supplementary Table S11. At Lag7, the OR for elevated SII associated with co-exposure was 1.109 (95% CI: 1.031–1.194).

## 4 Discussion

Based on outpatient data from a subtropical megacity, this study investigated the associations between co-exposure to extreme cold and NO_2_ and the risk of psoriasis outpatient visits, as well as exploratory analysis of systemic inflammatory markers in patients who underwent CBC tests. Our findings demonstrated that co-exposure was associated with increased risk of psoriasis outpatient visits at Lag14, with point estimates higher than those for single-factor exposures, although the additive interaction, as measured by RERI, was not statistically significant at Lag14. Stratified analyses indicated that the co-exposure effect was more pronounced in younger age groups. In exploratory analyses, co-exposure to extreme cold and NO_2_ was significantly associated with high SII levels at Lag7.

Despite the common clinical observation that psoriasis worsens in winter and improves in summer, epidemiological evidence on the role of temperature remains inconsistent. Some studies have found that decreasing temperatures are independently associated with reduced therapeutic efficacy (38), while a systematic review reported substantial heterogeneity, with 50% of psoriasis patients experiencing no seasonal variation, and 20% improving in winter (39). This inconsistency may stem from regional variations in air pollution levels. Several epidemiological studies have confirmed that short-term NO_2_ exposure significantly increases the risk of psoriasis outpatient visits (11–13). Notably, studies by Lan et al. (13) and Qu et al. (11) both observed that this risk-elevating effect is more pronounced in winter-a period when both low temperatures and elevated NO_2_ frequently co-occur. In fact, extreme cold and high NO_2_ concentrations often co-occur during winter. In our data, the RR for co-exposure at Lag14 was higher than that for single-factor exposures, suggesting a descriptive elevation in risk when both extreme cold and high NO_2_ occurred concurrently. However, the formal test for additive interaction at the primary Lag14 yielded a non-significant RERI (0.026, 95% CI: −0.055 to 0.108), isolated significant positive interactions were observed at Lag6 for T10_N90 and at Lag5 for T5_N95 (Supplementary Table S5); these were non-primary findings that were not consistently observed across thresholds or lag periods. Extreme cold disrupts the skin barrier function by reducing skin blood flow and increasing transepidermal water loss (15), and may activate the hypothalamic–pituitary–adrenal (HPA) axis and the sympathetic nervous system, leading to the release of pro-inflammatory cytokines, such as IL-6 and TNF-α (40, 41). Similarly, NO_2_ can induce oxidative stress by generating ROS (42), activating NF-κB signaling pathways and promotes pro-inflammatory cytokine production (43). However, our findings do not support a synergistic amplification in terms of outpatient visits. One possible explanation for the absence of synergistic interaction is the relatively low NO_2_ concentration during our study period, with a mean level of 37.6 μg/m^3^, which is lower than the 47.4 μg/m^3^ reported in a previous study conducted during 2015 to 2019 (13). This decline reflects the steady improvement in air quality in our study city, driven by the adoption of new energy vehicles and reductions in industrial emissions (44). The relatively low NO_2_ levels may have fallen below the threshold required to exert a measurable synergistic effect in combination with cold exposure, which is consistent with the null association observed for NO_2_ alone at Lag14. Future studies with larger sample sizes and longer time series are needed to further explore potential combined effects under varying pollution levels.

Previous studies on NO_2_ exposure in psoriasis reported higher risks in women, though formal interaction tests were often lacking (11–13). In our data, the RR for co-exposure was slightly higher in males than in females, but the interaction was not significant (*p* = 0.314), indicating no sex-specific effect modification. This suggests that sex is not a decisive modifier of the co-exposure effect in our study. The discrepancy with previous findings may be attributable to differences in exposure assessment, as prior studies focused on single-pollutant effects rather than combined environmental exposures (12, 13). Furthermore, in a modern urban setting, daily activity patterns, commuting behaviors, and time spent outdoors have become increasingly similar between men and women, potentially reducing differential exposure to ambient environmental factors, thereby attenuating the sex-specific susceptibility (45). In contrast, a clear age gradient was observed in our data. The RR was highest in the < 18 age group and gradually decreased with advancing age (*Q*-test, *p* = 0.004). This gradient may reflect age-related differences in both healthcare-seeking behavior and exposure patterns. Parents of children with psoriasis tend to seek medical care promptly, driven both by heightened concern over their children’s health and by the fact that pediatric patients are often experiencing first-time onset, which naturally prompts immediate attention (46). Conversely, working-age adults may delay medical visits due to competing work schedules, opportunity costs of time, higher tolerance for mild-to-moderate symptoms, or established coping habits for chronic conditions (47). Such delays would weaken the observed acute exposure-response associations in this age group. Older adults may have regular follow-up schedules and established disease management routines and may also show less reactive visit patterns to acute environmental triggers (48). Furthermore, younger individuals, particularly children, adolescents and young-to-middle-aged adults tend to have more outdoor activities, longer commutes, or outdoor occupations, leading to higher actual personal exposure doses to cold air and air pollution compared to older populations who are more likely to remain indoors (49). This higher exposure burden, combined with more timely healthcare-seeking behavior in the youngest age group, may jointly contribute to the elevated risk observed among individuals younger than 18 years.

Despite increasing evidence connecting low temperatures and air pollution to psoriasis, the mechanisms remain unclear. In our exploratory analysis of systemic inflammatory markers, the global tests for NLR and PLR were non-significant (*p* = 0.331 and 0.834, respectively). For SII, the global test was significant (*p* = 0.016), with an elevated estimate observed at Lag7 (OR = 1.109, 95% CI: 1.031–1.194). NO_2_ is a strong oxidizing gas that can cause epidermal oxidative damage, trigger inflammatory responses, disrupt the skin microbiota, and damage the skin barrier function, thereby increasing the risk of pathogen colonization and exacerbating psoriatic inflammation (43, 50, 51). Extreme cold can inhibit skin barrier repair and promote the release of pro-inflammatory cytokines (14, 16). When these two factors coexist, cold-induced barrier disruption could render the skin more vulnerable to NO_2_-mediated oxidative injury, potentially creating a synergistic local inflammatory environment that might contribute to psoriasis flare-up. However, this finding warrants cautious interpretation. Although SII showed a significant association, NLR and PLR did not, and multiple testing precluded formal correction of these exploratory analyses. Several factors may further explain the inconsistent results across markers and the modest effect estimate observed. The inflammatory markers assessed in this study reflect systemic rather than local cutaneous inflammation, and may not capture the localized skin responses that are most relevant to psoriasis pathophysiology. In addition, the relatively low NO_2_ levels during our study period may have been insufficient to trigger the hypothesized synergistic pathways. Moreover, the timing of blood sampling in routine clinical practice may not align optimally with the acute exposure window, potentially missing transient inflammatory responses that could have occurred closer to the exposure event.

Several limitations of this study should be acknowledged. First, data were collected from a single hospital, and although this institution is highly representative in Nanjing, the possibility of selection bias cannot be excluded. Second, due to the unavailability of individual residential addresses and related personal information, the use of city-wide average meteorological and air pollutant data inevitably introduces exposure measurement errors, as these average values cannot capture individual-level variability in residential location, occupation, commuting patterns, indoor heating exposure, or time spent outdoors. Third, hematological markers are typically tested in patients experiencing acute exacerbations, possessing extensive skin lesions, or those preparing for systemic medication and biologic therapies (52). Consequently, the data in our subgroup analysis may possess a selection bias, and these findings may not be generalizable to patients with mild psoriasis. Fourth, we lack validated clinical assessment indicators for the severity of psoriasis, such as PASI or BSA. Moreover, we are unable to adjust for individual-level confounding, including lifestyle, treatment history, or comorbidities, which may lead to residual confounding. Additionally, seasonal fluctuations and respiratory infections, which are known triggers for psoriasis flares, have not been directly considered, potentially introducing additional residual confounding factors. Fifth, outpatient visits are used as a proxy indicator for the exacerbation of psoriasis, but this indicator may be influenced by factors such as the behavior of seeking medical treatment, the accessibility of appointments, and the patient’s willingness to visit the doctor. It cannot fully reflect the actual disease activity or severity at the individual level. Finally, this study is based solely on clinical data from Nanjing. As an important industrial base and transportation hub in East China, Nanjing exhibits a typical northern subtropical monsoon climate and megacity pollution characteristics. Therefore, caution should be exercised when extrapolating the conclusions of this study to other climatic zones or regions with different pollution profiles. Future studies with larger sample sizes and individual-level data on exposure, clinical severity, and potential confounders are warranted to validate our findings and further elucidate the underlying mechanisms.

## 5 Conclusion

In this time series study, co-exposure to extreme cold and NO_2_ was associated with an increase in psoriasis outpatient visits at Lag14. Formal testing for additive interaction yielded non-significant, indicating no evidence of a synergistic effect between extreme cold and NO_2_. Stratified analyses revealed that the effect was more pronounced in younger age groups, with the highest RR observed in < 18 years, while no significant sex-specific effect modification was detected. In exploratory analyses, SII showed a significant global association with an elevated estimate at Lag7, whereas NLR and PLR did not reach statistical significance. These findings suggest that the observed co-exposure effect is modest and may be influenced by healthcare seeking behavior and residual confounding. The exploratory SII finding, while consistent with the hypothesized inflammatory pathway, should be interpreted cautiously and warrants confirmation in future mechanistic studies. Nonetheless, our results highlight the importance of considering healthcare accessibility and age-specific vulnerabilities when developing targeted public health interventions for psoriasis patients during extreme weather events.

## Data Availability

The original contributions presented in the study are included in the article/Supplementary material, further inquiries can be directed to the corresponding authors.

## Footnotes

1
https://data.cma.cn

2
https://air.cnemc.cn:18007/

## References

[1] Armstrong AW Read C. Pathophysiology, clinical presentation, and treatment of psoriasis: a review. JAMA. (2020) 323:1945–60. doi: 10.1001/jama.2020.4006, 32427307

[2] Armstrong AW Blauvelt A Callis Duffin K Huang YH Savage LJ Guo L . The Psoriasis. Nat Rev Dis Primers. (2025) 11:45. doi: 10.1038/s41572-025-00630-5 40571687

[3] Yang H Yang B Ye P Ho HC Li C Zhao W . Regional and local burden of psoriasis in China, 1990-2021: findings from the global burden of disease study 2021. Chin Med J. (1990) 139:314–6. doi: 10.1097/cm9.0000000000003636, 40654007 PMC12815521

[4] Puig L Ferrándiz C Pujol RM Vela E Albertí-Casas C Comellas M . Burden of psoriasis in Catalonia: epidemiology, associated comorbidities, health care utilization, and sick leave. Actas Dermosifiliogr (Engl Ed). (2021) 112:425–33. doi: 10.1016/j.ad.2020.11.017 33290733

[5] Wei J Wang Y Chen Y Wang Z Dai X Gelfand JM . Global burden of psoriasis from 1990 to 2021 and potential factors: a systematic analysis. J Invest Dermatol. (2026) 146:1034–45.e22. doi: 10.1016/j.jid.2025.08.038, 40930460

[6] Liu S He M Jiang J Duan X Chai B Zhang J . Triggers for the onset and recurrence of psoriasis: a review and update. Cell Commun Signal. (2024) 22:108. doi: 10.1186/s12964-023-01381-0, 38347543 PMC10860266

[7] Wu J Ma Y Yang J Tian Y. Exposure to air pollution, genetic susceptibility, and psoriasis risk in the UK. JAMA Netw Open. (2024) 7:e2421665. doi: 10.1001/jamanetworkopen.2024.21665, 39012635 PMC11252902

[8] Xiong Y Xia Y Zhang X Jiang B Zhang Z Xie C . Joint exposure to multiple air pollutants, genetic risk and incident psoriasis: a large-scale prospective cohort study. Br J Dermatol. (2025) 192:420–9. doi: 10.1093/bjd/ljae391, 39395185

[9] World Health Organization WHO global air quality guidelines: particulate matter (PM2.5 and PM10), ozone, nitrogen dioxide, sulfur dioxide and carbon monoxide (2021) Available online at: https://www.who.int/publications/i/item/9789240034228/ (Accessed July 21, 2026). 34662007

[10] Bosson JA Mudway IS Sandstrom T. Traffic-related air pollution, health, and allergy: the role of nitrogen dioxide. Am J Respir Crit Care Med. (2019) 200:523–4. doi: 10.1164/rccm.201904-0834ED, 31059649 PMC6727158

[11] Qu F Ma H Wang X Dong J Zhang J. Association between short-term air pollutants and psoriasis outpatient visits: a time series study in Nanchang, China. Int J Biometeorol. (2025) 69:2601–14. doi: 10.1007/s00484-025-02977-6, 40590951

[12] Wang T Xia Y Zhang X Qiao N Ke S Fang Q . Short-term effects of air pollutants on outpatients with psoriasis in a Chinese city with a subtropical monsoon climate. Front Public Health. (2022) 10:1071263. doi: 10.3389/fpubh.2022.1071263, 36620227 PMC9817471

[13] Lan J Huang Q Yang L Li Y Yang J Jiang B . Effects of ambient air pollution on outpatient visits for psoriasis in Wuhan, China: a time-series analysis. Br J Dermatol. (2023) 188:491–8. doi: 10.1093/bjd/ljac124, 36641781

[14] Hui-Beckman JW Goleva E Leung DYM Kim BE. The impact of temperature on the skin barrier and atopic dermatitis. Ann Allergy Asthma Immunol. (2023) 131:713–9. doi: 10.1016/j.anai.2023.08.007, 37595740

[15] Yang J Li G Yue L Dang E Qiao P. The impacts of seasonal factors on psoriasis. Exp Dermatol. (2025) 34:e70078. doi: 10.1111/exd.70078, 40103264

[16] Li FXZ Liu JJ Lei LM Li YH Xu F Lin X . Mechanism of cold exposure delaying wound healing in mice. J Nanobiotechnol. (2024) 22:723. doi: 10.1186/s12951-024-03009-y PMC11577949 39568002

[17] Wang J Wang S Xu X Li X He P Qiao Y . The diminishing effects of winter heating on air quality in northern China. J Environ Manag. (2023) 325:116536. doi: 10.1016/j.jenvman.2022.116536, 36326523

[18] Zhang Y Bo H Jiang Z Wang Y Fu Y Cao B . Untangling the contributions of meteorological conditions and human mobility to tropospheric NO(2) in Chinese mainland during the COVID-19 pandemic in early 2020. Natl Sci Rev. (2021) 8:nwab061. doi: 10.1093/nsr/nwab061, 34873447 PMC8083328

[19] Rahman MM McConnell R Schlaerth H Ko J Silva S Lurmann FW . The effects of coexposure to extremes of heat and particulate air pollution on mortality in California: implications for climate change. Am J Respir Crit Care Med. (2022) 206:1117–27. doi: 10.1164/rccm.202204-0657OC, 35727303 PMC9704834

[20] Ren H Xia Y Zhuang T Li Y Chen Y Huang W . Exposure to consecutive extreme ozone-heatwave events and neurological disorders: a retrospective cohort study in Nanjing, China. Environ Health. (2025) 24:74. doi: 10.1186/s12940-025-01234-y, 41074155 PMC12512602

[21] Zhou G Ren X Tang Z Li W Chen W He Y . Exploring the association and causal effect between white blood cells and psoriasis using large-scale population data. Front Immunol. (2023) 14:1043380. doi: 10.3389/fimmu.2023.1043380, 36865550 PMC9971993

[22] Liu YC Chuang SH Chen YP Shih YH. Associations of novel complete blood count-derived inflammatory markers with psoriasis: a systematic review and meta-analysis. Arch Dermatol Res. (2024) 316:228. doi: 10.1007/s00403-024-02994-2, 38787437

[23] Kimak-Pielas A Robak E Zajdel R Żebrowska A. The relationship between neutrophil-to-lymphocyte ratio, platelet-to-lymphocyte ratio, and systemic immune-inflammation index markers and response to biological therapy in patients with psoriasis. Int J Mol Sci. (2025) 26:3868. doi: 10.3390/ijms26083868, 40332589 PMC12028229

[24] Morariu SH Cotoi OS Tiucă OM Baican A Gheucă-Solovăstru L Decean H . Blood-count-derived inflammatory markers as predictors of response to biologics and small-molecule inhibitors in psoriasis: a multicenter study. J Clin Med. (2024) 13:3992. doi: 10.3390/jcm13143992, 39064032 PMC11277525

[25] Schneeweiss MC Shay D Ly S Wyss R Schneeweiss S Glynn RJ . Prevalence of pretreatment testing recommended for patients with chronic inflammatory skin diseases. JAMA Dermatol. (2024) 160:334–40. doi: 10.1001/jamadermatol.2023.5895, 38294794 PMC10831628

[26] Qiu W He H Wang B Wang D Mu G Xu T . Short-term impacts of air pollution on the platelet-lymphocyte ratio and neutrophil-lymphocyte ratio among urban adults in China. J Environ Sci (China). (2023) 125:101–11. doi: 10.1016/j.jes.2021.10.022, 36375897

[27] Yang Y Wu H Zeng Y Xu F Zhao S Zhang L . Short-term exposure to air pollution on peripheral white blood cells and inflammation biomarkers: a cross-sectional study on rural residents. BMC Public Health. (2024) 24:1702. doi: 10.1186/s12889-024-19116-2, 38926692 PMC11201365

[28] Qiu W He H Fan L Feng X Li M Dong C . Ambient temperature exposure causes lung function impairment: the evidence from controlled temperature study in healthy subjects (CTSHS). Int J Hyg Environ Health. (2023) 252:114214. doi: 10.1016/j.ijheh.2023.114214, 37392524

[29] Wang SW Tang KQ Zhang HR Liu WW Bai L Li N. Effect of carbon dioxide emission reduction policy on air quality improvement in Jiangsu Province. Huan Jing Ke Xue. (2023) 44:5443–55. doi: 10.13227/j.hjkx.202210203, 37827762

[30] Alahmad B Khraishah H Royé D Vicedo-Cabrera AM Guo Y Papatheodorou SI . Associations between extreme temperatures and cardiovascular cause-specific mortality: results from 27 countries. Circulation. (2023) 147:35–46. doi: 10.1161/circulationaha.122.061832, 36503273 PMC9794133

[31] Islam MM Satici MO Eroglu SE. Unraveling the clinical significance and prognostic value of the neutrophil-to-lymphocyte ratio, platelet-to-lymphocyte ratio, systemic immune-inflammation index, systemic inflammation response index, and delta neutrophil index: an extensive literature review. Turk J Emerg Med. (2024) 24:8–19. doi: 10.4103/tjem.tjem_198_23 PMC10852137 38343523

[32] Zinellu A Zinellu E Mangoni AA Pau MC Carru C Pirina P . Clinical significance of the neutrophil-to-lymphocyte ratio and platelet-to-lymphocyte ratio in acute exacerbations of COPD: present and future. Eur Respir Rev. (2022) 31:220095. doi: 10.1183/16000617.0095-2022, 36323421 PMC9724880

[33] Gasparrini A. Distributed lag linear and non-linear models in R: the package dlnm. J Stat Softw. (2011) 43:1–20. doi: 10.18637/jss.v043.i08, 22003319 PMC3191524

[34] Jiang Z Ji X Zhuo Y Hu J Chen S Xiang H . Mortality risk and burden attributable to compound cold extreme in China: a national time series study. Environ Int. (2025) 197:109364. doi: 10.1016/j.envint.2025.109364, 40090041

[35] Nagy B Pál-Jakab Á Kiss B Morvai A Sipos B Pápai G . Influence of extreme temperatures on out-of-hospital cardiac arrest cases in Hungary: a national time-series analysis. Resusc Plus. (2026) 27:101194. doi: 10.1016/j.resplu.2025.101194, 41583927 PMC12828408

[36] Zhang X Yang F Zhang J Dai Q. Using GAMs to explore the influence factors and their interactions on land surface temperature: a case study in Nanjing. Land (Basel). (2024) 13:465. doi: 10.3390/land13040465

[37] Noor AAM Lambuk L Ahmad F Azlan M Redzwan NM. Role of dendritic cells and B cells in the skin of imiquimod (IMQ)-induced psoriasis-like mouse model. PeerJ. (2026) 14:e20974. doi: 10.7717/peerj.20974 PMC13045838 41940392

[38] Song X Yang Q Wang Y Yu N. Impact of temperature trend-defined seasonality on psoriasis treatment outcomes: a multicenter longitudinal study. Front Immunol. (2025) 16:1641225. doi: 10.3389/fimmu.2025.1641225, 41041341 PMC12484154

[39] Jensen KK Serup J Alsing KK. Psoriasis and seasonal variation: a systematic review on reports from northern and Central Europe-little overall variation but distinctive subsets with improvement in summer or wintertime. Skin Res Technol. (2022) 28:180–6. doi: 10.1111/srt.13102, 34758175 PMC9907615

[40] Marek-Jozefowicz L Czajkowski R Borkowska A Nedoszytko B Żmijewski MA Cubała WJ . The brain-skin Axis in psoriasis-psychological, psychiatric, hormonal, and dermatological aspects. Int J Mol Sci. (2022) 23:669. doi: 10.3390/ijms23020669, 35054853 PMC8776235

[41] Lei D Gong C Wang B Zhang L Zhang G Man MQ. The role of psychological stress in the pathogenesis of psoriasis. Front Med (Lausanne). (2025) 12:1614863. doi: 10.3389/fmed.2025.1614863, 40861201 PMC12375474

[42] Yan W Ji X Shi J Li G Sang N. Acute nitrogen dioxide inhalation induces mitochondrial dysfunction in rat brain. Environ Res. (2015) 138:416–24. doi: 10.1016/j.envres.2015.02.022, 25791864

[43] Plenkowska J Gabig-Ciminska M Mozolewski P. Oxidative stress as an important contributor to the pathogenesis of psoriasis. Int J Mol Sci. (2020) 21:6206. doi: 10.3390/ijms21176206, 32867343 PMC7503883

[44] Javed Z Bilal M Qiu Z Li G Sandhu O Mehmood K . Spatiotemporal characterization of aerosols and trace gases over the Yangtze River Delta region, China: impact of trans-boundary pollution and meteorology. Environ Sci Eur. (2022) 34:86. doi: 10.1186/s12302-022-00668-2, 36097441 PMC9453706

[45] Kwon K Akar G. Have the gender differences in commuting been shrinking or persistent? Evidence from two-earner households in the U.S. Int J Sustain Transp. (2022) 16:1121–30. doi: 10.1080/15568318.2021.1971345

[46] Dhar S Srinivas SM. Psoriasis in pediatric age group. Indian J Dermatol. (2022) 67:374–80. doi: 10.4103/ijd.ijd_570_22, 36578742 PMC9792015

[47] Thorneloe RJ Bundy C Griffiths CE Ashcroft DM Cordingley L. Nonadherence to psoriasis medication as an outcome of limited coping resources and conflicting goals: findings from a qualitative interview study with people with psoriasis. Br J Dermatol. (2017) 176:667–76. doi: 10.1111/bjd.15086, 27664406 PMC5363250

[48] Zhang Y Yan C Kan H Cao J Peng L Xu J . Effect of ambient temperature on emergency department visits in Shanghai, China: a time series study. Environ Health. (2014) 13:100. doi: 10.1186/1476-069x-13-100, 25424196 PMC4258028

[49] European Environment Agency Air pollution and children's health (2023) Available online at:https://www.eea.europa.eu/en/analysis/publications/air-pollution-and-childrens-health (Accessed July 27, 2026).

[50] Janvier X Alexandre S Boukerb AM Souak D Maillot O Barreau M . Deleterious effects of an air pollutant (NO(2)) on a selection of commensal skin bacterial strains, potential contributor to Dysbiosis? Front Microbiol. (2020) 11:591839. doi: 10.3389/fmicb.2020.591839, 33363523 PMC7752777

[51] Chang HW Yan D Singh R Liu J Lu X Ucmak D . Alteration of the cutaneous microbiome in psoriasis and potential role in Th17 polarization. Microbiome. (2018) 6:154. doi: 10.1186/s40168-018-0533-1, 30185226 PMC6125946

[52] Fenkel JM Osborne JA Armstrong AW Strober B Stein Gold L Cameron M . Laboratory monitoring in patients with moderate to severe plaque psoriasis. J Dermatolog Treat. (2025) 36:2583227. doi: 10.1080/09546634.2025.2583227, 41346021

