# Supplementary material for: Association of co-exposure to extreme cold and nitrogen dioxide with psoriasis outpatient visits and systemic inflammation
Source: Front Public Health. 2026 Sep 9;14:1916342. doi: 10.3389/fpubh.2026.1916342 (PMC13597943; doi:10.3389/fpubh.2026.1916342)
Supplement: Supplementary file 1 [file Table_1.docx]

***Supplementary Material***

**Supplementary tables**

**Table S1.** Analysis of the correlation between meteorological and air pollutants data in Nanjing (November-March, 2021-2025).

**Table S2.** Concurvity (worst-case) for smooth terms and the variance inflation factor for parametric terms for the main model.

**Table S3.** Number of extreme exposure days, corresponding outpatient visits, and model diagnostics by exposure threshold (November-March, 2021-2025).

**Table S4.** Single-day, cumulative lag relative risks [RR(95%CI)] and the global *P* value of extreme cold, NO_2_ and co-exposure on the outpatient visits for psoriasis by different threshold.

**Table S5.** Single-day relative excess risk due to interaction of co-exposure on the outpatient visits for psoriasis by different threshold. [RERI(95%CI)]

**Table S6.** Single-day relative risks of T10_N90 on the outpatient visits for psoriasis in subgroup analysis by sex. [RR(95%CI)]

**Table S7.** Single-day relative risks of T10_N90 on the outpatient visits for psoriasis in subgroup analysis by age. [RR(95%CI)]

**Table S8.** Single-day Lag14 relative risks of T10, N90 and T10_N90 in sensitivity analysis by different model specifications: main model, 14-day washout, and 28-day washout. [RR(95%CI)]

**Table S9.** Single-day Lag14 relative risks of exposure to T10, N90 and T10_N90 on the outpatient visits for psoriasis in sensitivity analysis by changing air pollutants. [RR(95%CI)]

**Table S10.** Single-day Lag14 relative risks of T10, N90 and T10_N90 on the outpatient visits for psoriasis in sensitivity analysis by changing the *k* of time. [RR(95%CI)]

**Table S11.** Single-day odds ratios [OR(95%CI)] and the global *P* value of T10_N90 on the high systemic inflammation of psoriasis in the outpatient visits.

**Table S1.** Analysis of the correlation between meteorological and air pollutants data in Nanjing (November-March, 2021-2025).

|  | **Tmean** | **RH** | **PM_10_** | **PM_2.5_** | **NO_2_** | **SO_2_** | **O_3_** | **CO** |
| --- | --- | --- | --- | --- | --- | --- | --- | --- |
| Tmean | 1.000 | **.176** | **.135** | 0.032 | **.117** | -0.001 | **.327** | **.082** |
| RH |  | 1.000 | **-.214** | 0.061 | **-.083** | **-.423** | **-.179** | -0.007 |
| PM_10_ |  |  | 1.000 | **.822** | **.585** | **.337** | -0.060 | **.508** |
| PM_2.5_ |  |  |  | 1.000 | **.561** | **.242** | **-.092** | **.570** |
| NO_2_ |  |  |  |  | 1.000 | **.393** | **-.471** | **.582** |
| SO_2_ |  |  |  |  |  | 1.000 | 0.012 | **.351** |
| O_3_ |  |  |  |  |  |  | 1.000 | **-.247** |
| CO |  |  |  |  |  |  |  | 1.000 |

Tmean, daily mean temperature; RH, daily relative humidity; PM_2.5_, particulate matter with an aerodynamic diameter of 2.5 μm or less; PM_10_, particulate matter with an aerodynamic diameter of less than 10 μm; NO_2_, nitrogen dioxide; SO_2_, sulfur dioxide; CO, carbon monoxide; O3, ozone. Values in bold are statistically significant at the *P* < 0.05 level.

**Table S2.** Concurvity (worst-case) for smooth terms and the variance inflation factor for parametric terms for the main model.

| **Concurvity for smooth terms** | | **Variance inflation factor for parametric terms** | |
| --- | --- | --- | --- |
| **Smooth term** | **Worst concurvity** | **Parametric term** | **VIF** |
| s(RH) | 0.174 | NO_2_ | 2.001 |
| s(PM_2.5_) | 0.513 | SO_2_ | 1.381 |
| s(CO) | 0.464 | O_3_ | 1.331 |
| s(Time) | 0.177 | CO | 1.651 |

VIF, Variance inflation factors; RH, daily relative humidity; PM_2.5_, particulate matter with an aerodynamic diameter of 2.5 μm or less; NO_2_, nitrogen dioxide; SO_2_, sulfur dioxide; CO, carbon monoxide; O3, ozone. Concurvity values < 0.8 indicate no serious concurvity. To assess potential multicollinearity among the air pollutants (PM_2.5_, NO_2_, SO_2_, O_3_, and CO), we calculated variance inflation factors (VIF) using a multiple linear regression model incorporating all concurrent pollutants. VIF values < 5 indicating that severe multicollinearity was absent.

**Table S3.** Number of extreme exposure days, corresponding outpatient visits, and model diagnostics by exposure threshold (November-March, 2021-2025).

| **Definition** | **Threshold** | **Value** | **Exposure days (n)** | **Outpatient visits (n)** | **Dispersion parameter (*θ*)** | **AIC** |
| --- | --- | --- | --- | --- | --- | --- |
| Extreme cold |  |  |  |  |  |  |
| T10 | Tmean ≤ 10% | Tmean ≤ 6.1℃ | 188 | 14669 | 48.66 | 4474.783 |
| T7.5 | Tmean ≤ 7.5% | Tmean ≤ 5.4℃ | 136 | 10299 | 47.53 | 4481.521 |
| T5 | Tmean ≤ 5% | Tmean ≤ 4℃ | 91 | 6543 | 48.32 | 4477.134 |
| Extreme NO_2_ |  |  |  |  |  |  |
| N90 | NO_2_ ≥ 90% | NO_2_ ≥ 42μg/m^3^ | 177 | 15826 | 47.96 | 4479.398 |
| N92.5 | NO_2_ ≥ 92.5% | NO_2_ ≥ 45μg/m^3^ | 130 | 11809 | 46.81 | 4486.758 |
| N95 | NO_2_ ≥ 95% | NO_2_ ≥ 50μg/m^3^ | 85 | 7673 | 47.63 | 4481.516 |
| Co-exposure |  |  |  |  |  |  |
| T10_N90 | Tmean ≤ 10% and NO_2_ ≥ 90% | Tmean ≤ 6.1℃ and NO_2_ ≥ 42μg/m^3^ | 40 | 3101 | 47.10 | 4484.984 |
| T7.5_N92.5 | Tmean ≤ 7.5% and NO_2_ ≥ 92.5% | Tmean ≤ 5.4℃ and NO_2_ ≥ 45μg/m^3^ | 18 | 1511 | 47.09 | 4484.916 |
| T5_N95 | Tmean ≤ 5% and NO_2_ ≥ 95% | Tmean ≤ 4℃ and NO_2_ ≥ 50μg/m^3^ | 6 | 548 | 48.32 | 4477.922 |

AIC, Akaike Information Criterion; Tmean, daily mean temperature; NO_2_, nitrogen dioxide; n, number. The effective sample size (the total number of days) during the study period (November-March, 2021-2025) was 756 days.

**Table S4.** Single-day, cumulative lag relative risks [RR(95%CI)] and the global *P* value of extreme cold, NO_2_ and co-exposure on the outpatient visits for psoriasis by different threshold.

| **Lag** | **T10** | **N90** | **T10_N90** | **T7.5** | **N92.5** | **T7.5_N92.5** | **T5** | **N95** | **T5_N95** |
| --- | --- | --- | --- | --- | --- | --- | --- | --- | --- |
| Single-day lag effect | | | | | | | | | |
| 0 | **0.983(0.970,0.996)** | 0.993(0.975,1.011) | 0.981(0.952,1.010) | **0.984(0.970,0.998)** | 1.004(0.984,1.025) | 1.007(0.960,1.056) | 0.986(0.969,1.004) | 0.999(0.977,1.021) | 1.017(0.944,1.096) |
| 1 | **0.986(0.975,0.997)** | 0.992(0.977,1.007) | 0.983(0.959,1.008) | **0.987(0.975,0.999)** | 1.003(0.985,1.021) | 1.007(0.967,1.050) | 0.989(0.975,1.004) | 0.997(0.978,1.017) | 1.018(0.954,1.086) |
| 2 | **0.989(0.980,0.999)** | 0.992(0.979,1.004) | 0.986(0.966,1.007) | 0.991(0.981,1.001) | 1.001(0.987,1.016) | 1.008(0.973,1.044) | 0.992(0.980,1.004) | 0.996(0.980,1.012) | 1.019(0.964,1.077) |
| 3 | 0.993(0.985,1.000) | 0.991(0.981,1.002) | 0.989(0.972,1.006) | 0.994(0.986,1.003) | 1.000(0.988,1.012) | 1.009(0.979,1.040) | 0.995(0.985,1.005) | 0.994(0.981,1.008) | 1.020(0.973,1.069) |
| 4 | 0.996(0.989,1.002) | 0.991(0.983,1.000) | 0.991(0.977,1.006) | 0.998(0.991,1.005) | 0.999(0.989,1.009) | 1.010(0.984,1.037) | 0.998(0.990,1.006) | 0.993(0.982,1.005) | 1.021(0.979,1.064) |
| 5 | 0.999(0.993,1.004) | **0.991(0.984,0.998)** | 0.994(0.981,1.007) | 1.001(0.994,1.007) | 0.998(0.989,1.007) | 1.011(0.987,1.036) | 1.000(0.993,1.008) | 0.992(0.982,1.002) | 1.022(0.983,1.063) |
| 6 | 1.001(0.996,1.007) | **0.991(0.984,0.998)** | 0.997(0.984,1.009) | 1.003(0.997,1.010) | 0.997(0.989,1.005) | 1.012(0.989,1.036) | 1.003(0.996,1.010) | 0.991(0.982,1.001) | 1.024(0.986,1.064) |
| 7 | 1.004(0.998,1.010) | **0.991(0.984,0.998)** | 0.999(0.986,1.013) | 1.006(0.999,1.013) | 0.996(0.988,1.004) | 1.014(0.990,1.038) | 1.005(0.997,1.013) | 0.991(0.981,1.001) | 1.026(0.986,1.067) |
| 8 | 1.006(1.000,1.012) | **0.992(0.984,0.999)** | 1.002(0.988,1.016) | **1.008(1.001,1.015)** | 0.996(0.987,1.004) | 1.015(0.990,1.040) | 1.007(0.998,1.015) | 0.991(0.980,1.001) | 1.028(0.987,1.072) |
| 9 | **1.008(1.001,1.015)** | 0.992(0.985,1.000) | 1.005(0.990,1.020) | **1.010(1.002,1.018)** | 0.996(0.987,1.005) | 1.016(0.991,1.042) | 1.008(0.999,1.017) | 0.991(0.980,1.002) | 1.031(0.988,1.077) |
| 10 | **1.009(1.002,1.017)** | 0.993(0.985,1.002) | 1.007(0.992,1.023) | **1.011(1.003,1.019)** | 0.996(0.986,1.005) | 1.018(0.992,1.045) | 1.009(0.999,1.018) | 0.991(0.980,1.003) | 1.035(0.990,1.082) |
| 11 | **1.010(1.003,1.018)** | 0.995(0.986,1.003) | 1.010(0.994,1.025) | **1.012(1.004,1.020)** | 0.996(0.987,1.006) | 1.020(0.994,1.046) | 1.009(1.000,1.019) | 0.992(0.981,1.004) | 1.039(0.994,1.086) |
| 12 | **1.011(1.004,1.018)** | 0.996(0.988,1.004) | 1.012(0.998,1.027) | **1.012(1.004,1.020)** | 0.997(0.988,1.006) | 1.022(0.997,1.047) | **1.009(1.000,1.018)** | 0.994(0.983,1.005) | 1.044(0.999,1.090) |
| 13 | **1.011(1.005,1.018)** | 0.998(0.991,1.006) | **1.015(1.001,1.029)** | **1.011(1.004,1.019)** | 0.998(0.990,1.007) | 1.024(1.000,1.048) | **1.009(1.000,1.017)** | 0.996(0.985,1.006) | **1.049(1.005,1.094)** |
| 14 | **1.011(1.005,1.017)** | 1.000(0.993,1.007) | **1.017(1.004,1.030)** | **1.010(1.004,1.017)** | 1.000(0.992,1.008) | **1.026(1.003,1.049)** | 1.008(1.000,1.016) | 0.998(0.988,1.008) | **1.055(1.012,1.099)** |
| 15 | **1.011(1.005,1.016)** | 1.002(0.996,1.009) | **1.020(1.007,1.032)** | **1.009(1.003,1.016)** | 1.001(0.993,1.009) | **1.028(1.006,1.051)** | 1.007(0.999,1.014) | 1.001(0.991,1.010) | **1.061(1.018,1.106)** |
| 16 | **1.010(1.004,1.016)** | 1.005(0.998,1.012) | **1.022(1.009,1.035)** | **1.008(1.002,1.014)** | 1.003(0.995,1.011) | **1.031(1.007,1.055)** | 1.005(0.998,1.012) | 1.003(0.994,1.013) | **1.067(1.022,1.115)** |
| 17 | **1.009(1.003,1.015)** | **1.008(1.000,1.015)** | **1.025(1.010,1.040)** | 1.006(0.999,1.013) | 1.005(0.996,1.014) | **1.033(1.006,1.061)** | 1.003(0.995,1.011) | 1.007(0.996,1.017) | **1.074(1.024,1.127)** |
| 18 | **1.008(1.001,1.015)** | **1.010(1.001,1.020)** | **1.027(1.009,1.046)** | 1.004(0.996,1.012) | 1.007(0.997,1.018) | **1.036(1.004,1.068)** | 1.001(0.992,1.011) | 1.010(0.998,1.022) | **1.082(1.023,1.143)** |
| 19 | 1.007(0.998,1.016) | **1.013(1.002,1.025)** | **1.029(1.007,1.052)** | 1.002(0.992,1.011) | 1.010(0.997,1.023) | **1.038(1.001,1.078)** | 0.999(0.988,1.011) | 1.013(0.999,1.028) | **1.089(1.021,1.161)** |
| 20 | 1.005(0.995,1.016) | **1.016(1.003,1.030)** | **1.032(1.005,1.060)** | 0.999(0.988,1.011) | 1.012(0.997,1.028) | 1.041(0.996,1.088) | 0.997(0.983,1.011) | 1.017(1.000,1.035) | **1.097(1.018,1.182)** |
| 21 | 1.004(0.991,1.017) | **1.019(1.003,1.036)** | **1.034(1.002,1.067)** | 0.997(0.983,1.011) | 1.015(0.996,1.033) | 1.044(0.992,1.099) | 0.995(0.979,1.011) | **1.021(1.000,1.041)** | **1.104(1.013,1.204)** |
| Cumulative lag effect | | | | | | | | | |
| 0-1 | **0.969(0.945,0.993)** | 0.985(0.953,1.018) | 0.964(0.913,1.019) | **0.971(0.946,0.997)** | 1.007(0.970,1.046) | 1.014(0.928,1.108) | 0.976(0.945,1.007) | 0.996(0.955,1.038) | 1.036(0.901,1.190) |
| 0-2 | **0.958(0.926,0.992)** | 0.977(0.933,1.022) | 0.951(0.882,1.025) | **0.962(0.928,0.998)** | 1.008(0.957,1.063) | 1.022(0.903,1.157) | 0.968(0.926,1.012) | 0.992(0.936,1.051) | 1.055(0.869,1.280) |
| 0-3 | **0.951(0.913,0.991)** | 0.968(0.916,1.024) | 0.940(0.858,1.031) | 0.957(0.915,1.000) | 1.009(0.946,1.076) | 1.032(0.886,1.202) | 0.963(0.913,1.016) | 0.986(0.919,1.059) | 1.076(0.847,1.366) |
| 0-4 | **0.947(0.904,0.993)** | 0.960(0.901,1.023) | 0.932(0.839,1.035) | 0.955(0.907,1.004) | 1.007(0.936,1.084) | 1.042(0.874,1.244) | 0.961(0.904,1.022) | 0.980(0.903,1.062) | 1.098(0.833,1.448) |
| 0-5 | **0.946(0.899,0.995)** | 0.951(0.887,1.019) | 0.926(0.826,1.039) | 0.955(0.904,1.010) | 1.005(0.927,1.090) | 1.054(0.866,1.283) | 0.962(0.900,1.028) | 0.972(0.889,1.063) | 1.123(0.826,1.528) |
| 0-6 | **0.947(0.897,1.000)** | 0.943(0.876,1.015) | 0.923(0.817,1.044) | 0.958(0.904,1.017) | 1.002(0.919,1.092) | 1.067(0.863,1.320) | 0.964(0.899,1.035) | 0.964(0.876,1.061) | 1.150(0.823,1.606) |
| 0-7 | 0.951(0.898,1.006) | 0.935(0.866,1.009) | 0.923(0.811,1.050) | 0.964(0.906,1.026) | 0.998(0.912,1.092) | 1.082(0.862,1.357) | 0.969(0.900,1.043) | 0.955(0.863,1.056) | 1.180(0.825,1.687) |
| 0-8 | 0.956(0.902,1.014) | 0.927(0.857,1.003) | 0.925(0.808,1.057) | 0.972(0.911,1.037) | 0.994(0.906,1.091) | 1.098(0.864,1.395) | 0.975(0.904,1.053) | 0.946(0.852,1.051) | 1.213(0.830,1.774) |
| 0-9 | 0.964(0.907,1.024) | **0.920(0.849,0.997)** | 0.929(0.808,1.067) | 0.982(0.918,1.049) | 0.989(0.900,1.088) | 1.116(0.868,1.434) | 0.983(0.909,1.064) | 0.937(0.841,1.045) | 1.251(0.837,1.870) |
| 0-10 | 0.973(0.914,1.035) | **0.914(0.842,0.991)** | 0.935(0.810,1.080) | 0.992(0.926,1.064) | 0.985(0.894,1.086) | 1.136(0.873,1.477) | 0.992(0.914,1.076) | 0.929(0.830,1.040) | 1.295(0.848,1.978) |
| 0-11 | 0.983(0.922,1.048) | **0.909(0.837,0.987)** | 0.944(0.814,1.096) | 1.004(0.934,1.079) | 0.981(0.889,1.083) | 1.158(0.880,1.524) | 1.001(0.920,1.090) | 0.922(0.821,1.036) | 1.345(0.860,2.103) |
| 0-12 | 0.994(0.930,1.062) | **0.905(0.832,0.985)** | 0.956(0.819,1.116) | 1.016(0.942,1.095) | 0.978(0.885,1.081) | 1.183(0.889,1.574) | 1.010(0.925,1.103) | 0.917(0.813,1.033) | 1.404(0.877,2.247) |
| 0-13 | 1.005(0.938,1.076) | **0.904(0.830,0.984)** | 0.970(0.827,1.138) | 1.027(0.950,1.110) | 0.977(0.882,1.081) | 1.211(0.901,1.629) | 1.019(0.930,1.117) | 0.913(0.807,1.032) | 1.472(0.898,2.413) |
| 0-14 | 1.016(0.947,1.090) | **0.904(0.829,0.985)** | 0.987(0.837,1.163) | 1.038(0.957,1.125) | 0.976(0.881,1.081) | 1.242(0.915,1.687) | 1.027(0.934,1.129) | 0.911(0.803,1.034) | 1.552(0.925,2.605) |
| 0-15 | 1.027(0.956,1.103) | **0.906(0.831,0.987)** | 1.006(0.850,1.190) | 1.047(0.964,1.138) | 0.977(0.882,1.083) | 1.277(0.933,1.750) | 1.034(0.938,1.140) | 0.912(0.802,1.037) | 1.647(0.959,2.826) |
| 0-16 | 1.037(0.964,1.115) | **0.910(0.836,0.992)** | 1.028(0.866,1.221) | 1.056(0.970,1.149) | 0.980(0.885,1.086) | 1.317(0.954,1.817) | 1.039(0.940,1.149) | 0.915(0.803,1.042) | **1.758(1.002,3.084)** |
| 0-17 | 1.046(0.972,1.126) | **0.917(0.843,0.998)** | 1.054(0.885,1.254) | 1.062(0.974,1.157) | 0.986(0.891,1.091) | 1.360(0.979,1.890) | 1.043(0.941,1.155) | 0.921(0.807,1.051) | **1.888(1.052,3.388)** |
| 0-18 | 1.055(0.980,1.135) | 0.927(0.852,1.008) | 1.082(0.906,1.292) | 1.066(0.977,1.163) | 0.993(0.898,1.098) | **1.409(1.007,1.971)** | 1.044(0.941,1.159) | 0.930(0.814,1.062) | **2.042(1.111,3.751)** |
| 0-19 | 1.062(0.986,1.143) | 0.939(0.864,1.020) | 1.114(0.929,1.336) | 1.068(0.978,1.166) | 1.003(0.907,1.108) | **1.463(1.036,2.065)** | 1.043(0.938,1.160) | 0.942(0.824,1.078) | **2.224(1.178,4.197)** |
| 0-20 | 1.068(0.990,1.151) | 0.954(0.878,1.037) | 1.149(0.952,1.388) | 1.067(0.976,1.167) | 1.015(0.918,1.122) | **1.523(1.066,2.176)** | 1.040(0.932,1.161) | 0.958(0.836,1.099) | **2.439(1.251,4.755)** |
| 0-21 | 1.072(0.992,1.159) | 0.973(0.892,1.060) | 1.189(0.974,1.451) | 1.064(0.970,1.167) | 1.030(0.928,1.143) | **1.589(1.093,2.311)** | 1.035(0.923,1.160) | 0.978(0.849,1.126) | **2.693(1.326,5.471)** |
| ^a^*P* | **0.002** | 0.054 | **0.008** | **0.019** | 0.469 | 0.065 | 0.233 | 0.196 | **0.028** |

RR, relative risk; RERI, relative excess risk due to interaction.^a^*P*, Global *P*‑values were derived from the joint Wald chi‑square test for the cross‑basis term, testing the joint significance of the exposure across all 21 lag days. Threshold definitions: T10: daily mean temperature ≤ 10th percentile (6.1 °C); N90: daily mean NO_2_ ≥ 90th percentile (42 μg/m^3^); T10_N90: both temperature ≤10th percentile and NO_2_ ≥ 90th percentile; T7.5: daily mean temperature ≤ 7.5th percentile (5.4 °C); N92.5: daily mean NO_2_ ≥ 92.5th percentile (45 μg/m^3^); T7.5_N92.5: both temperature ≤ 7.5th percentile and NO_2_ ≥ 92.5th percentile; T5: daily mean temperature ≤ 5th percentile (4 °C); N95: daily mean NO_2_ ≥ 95th percentile (50 μg/m^3^); T5_N95: both temperature ≤ 5th percentile and NO_2_ ≥ 95th percentile. Values in bold are statistically significant at the *P* < 0.05 level.

**Table S5.** Single-day relative excess risk due to interaction of co-exposure on the outpatient visits for psoriasis by different threshold. [RERI(95%CI)]

| **Lag** | **T10_N90** | **T7.5_N92.5** | **T5_N95** |
| --- | --- | --- | --- |
| 0 | -0.030(-0.106,0.045) | -0.038(-0.136,0.061) | -0.023(-0.171,0.124) |
| 1 | -0.026(-0.101,0.048) | 0.038(-0.066,0.142) | -0.001(-0.172,0.169) |
| 2 | -0.016(-0.092,0.061) | -0.010(-0.116,0.095) | -0.027(-0.222,0.167) |
| 3 | -0.042(-0.118,0.033) | -0.038(-0.141,0.065) | 0.003(-0.153,0.159) |
| 4 | 0.001(-0.075,0.077) | 0.100(-0.003,0.203) | 0.080(-0.081,0.242) |
| 5 | 0.034(-0.043,0.111) | 0.041(-0.061,0.142) | **0.171(0.005,0.336)** |
| 6 | **0.089(0.008,0.171)** | 0.020(-0.085,0.124) | 0.077(-0.087,0.240) |
| 7 | 0.047(-0.030,0.125) | 0.032(-0.072,0.136) | 0.064(-0.098,0.226) |
| 8 | -0.014(-0.091,0.063) | 0.020(-0.085,0.126) | 0.073(-0.095,0.242) |
| 9 | 0.001(-0.077,0.079) | -0.019(-0.126,0.088) | -0.028(-0.204,0.148) |
| 10 | -0.037(-0.116,0.042) | -0.018(-0.123,0.088) | 0.028(-0.145,0.201) |
| 11 | -0.011(-0.091,0.069) | 0.014(-0.089,0.116) | 0.067(-0.105,0.238) |
| 12 | -0.024(-0.106,0.059) | 0.002(-0.108,0.113) | -0.066(-0.228,0.096) |
| 13 | 0.023(-0.058,0.104) | -0.022(-0.128,0.084) | -0.093(-0.268,0.083) |
| 14 | 0.026(-0.055,0.108) | -0.052(-0.159,0.054) | -0.013(-0.200,0.173) |
| 15 | 0.038(-0.045,0.121) | 0.057(-0.054,0.168) | 0.143(-0.092,0.379) |
| 16 | -0.060(-0.144,0.023) | -0.091(-0.205,0.022) | 0.022(-0.244,0.288) |
| 17 | 0.050(-0.039,0.139) | -0.012(-0.136,0.112) | 0.003(-0.252,0.258) |
| 18 | 0.070(-0.018,0.158) | 0.085(-0.035,0.206) | 0.115(-0.112,0.342) |
| 19 | 0.019(-0.072,0.110) | 0.068(-0.064,0.200) | 0.187(-0.025,0.399) |
| 20 | 0.042(-0.049,0.133) | 0.065(-0.059,0.189) | 0.142(-0.097,0.380) |
| 21 | 0.080(-0.014,0.175) | 0.070(-0.060,0.200) | 0.112(-0.100,0.323) |

Threshold definitions: T10_N90: both temperature ≤10th percentile (6.1 °C) and NO_2_ ≥ 90th percentile (42 μg/m^3^); T7.5_N92.5: both temperature ≤ 7.5th percentile (5.4 °C) and NO_2_ ≥ 92.5th percentile (45 μg/m^3^); T5_N95: both temperature ≤ 5th percentile (4 °C) and NO_2_ ≥ 95th percentile (50 μg/m^3^). Values in bold are statistically significant at the *P* < 0.05 level.

**Table S6.** Single-day relative risks of T10_N90 on the outpatient visits for psoriasis in subgroup analysis by sex. [RR(95%CI)]

| **Lag** | **Male** | **Female** |
| --- | --- | --- |
| 0 | 0.984(0.954,1.015) | 0.973(0.937,1.011) |
| 1 | 0.987(0.961,1.013) | 0.974(0.943,1.006) |
| 2 | 0.990(0.969,1.012) | 0.976(0.950,1.002) |
| 3 | 0.993(0.976,1.012) | 0.977(0.955,0.999) |
| 4 | 0.997(0.981,1.012) | 0.978(0.960,0.997) |
| 5 | 1.000(0.986,1.013) | 0.980(0.963,0.997) |
| 6 | 1.003(0.989,1.016) | 0.982(0.966,0.998) |
| 7 | 1.005(0.992,1.019) | 0.984(0.967,1.001) |
| 8 | 1.008(0.993,1.023) | 0.986(0.968,1.004) |
| 9 | 1.011(0.995,1.026) | 0.989(0.970,1.008) |
| 10 | 1.013(0.997,1.029) | 0.992(0.973,1.012) |
| 11 | 1.015(0.999,1.031) | 0.996(0.977,1.015) |
| 12 | **1.017(1.002,1.032)** | 1.000(0.981,1.019) |
| 13 | **1.019(1.004,1.033)** | 1.004(0.987,1.022) |
| 14 | **1.020(1.007,1.034)** | 1.009(0.993,1.026) |
| 15 | **1.021(1.009,1.034)** | 1.014(0.999,1.030) |
| 16 | **1.023(1.009,1.036)** | **1.020(1.003,1.037)** |
| 17 | **1.024(1.008,1.040)** | **1.026(1.007,1.045)** |
| 18 | **1.025(1.006,1.044)** | **1.032(1.008,1.055)** |
| 19 | **1.026(1.002,1.050)** | **1.038(1.009,1.067)** |
| 20 | 1.027(0.998,1.056) | **1.044(1.009,1.080)** |
| 21 | 1.027(0.994,1.062) | **1.050(1.009,1.093)** |

Values in bold are statistically significant at the *P* < 0.05 level.

**Table S7.** Single-day relative risks of T10_N90 on the outpatient visits for psoriasis in subgroup analysis by age. [RR(95%CI)]

| **Lag** | < 18 years | 18-45 years | 46-65 years | > 65 years |
| --- | --- | --- | --- | --- |
| 0 | 0.912(0.799,1.041) | 0.963(0.928,1.000) | 0.999(0.965,1.035) | 0.973(0.925,1.024) |
| 1 | 0.919(0.822,1.029) | 0.969(0.939,1.000) | 1.000(0.971,1.030) | 0.975(0.934,1.018) |
| 2 | 0.927(0.844,1.018) | 0.975(0.950,1.001) | 1.000(0.976,1.025) | 0.977(0.943,1.013) |
| 3 | 0.934(0.864,1.010) | 0.981(0.960,1.002) | 1.001(0.981,1.021) | 0.979(0.950,1.009) |
| 4 | 0.942(0.882,1.006) | 0.987(0.969,1.005) | 1.001(0.984,1.018) | 0.981(0.957,1.006) |
| 5 | 0.950(0.897,1.007) | 0.992(0.976,1.008) | 1.002(0.986,1.017) | 0.983(0.961,1.005) |
| 6 | 0.959(0.907,1.014) | 0.998(0.982,1.014) | 1.002(0.987,1.017) | 0.984(0.963,1.006) |
| 7 | 0.968(0.914,1.024) | 1.003(0.987,1.020) | 1.003(0.987,1.019) | 0.985(0.963,1.009) |
| 8 | 0.977(0.920,1.038) | 1.008(0.991,1.026) | 1.004(0.987,1.020) | 0.986(0.962,1.011) |
| 9 | 0.987(0.926,1.052) | 1.013(0.995,1.032) | 1.005(0.987,1.022) | 0.987(0.962,1.013) |
| 10 | 0.997(0.934,1.065) | 1.018(0.999,1.037) | 1.006(0.988,1.024) | 0.988(0.961,1.014) |
| 11 | 1.009(0.945,1.076) | **1.022(1.003,1.041)** | 1.007(0.989,1.025) | 0.987(0.962,1.014) |
| 12 | 1.021(0.959,1.086) | **1.026(1.007,1.044)** | 1.008(0.991,1.026) | 0.987(0.962,1.012) |
| 13 | 1.033(0.975,1.095) | **1.029(1.012,1.047)** | 1.010(0.994,1.026) | 0.986(0.963,1.010) |
| 14 | 1.047(0.992,1.104) | **1.033(1.017,1.049)** | 1.012(0.997,1.027) | 0.985(0.964,1.007) |
| 15 | **1.060(1.008,1.115)** | **1.036(1.020,1.051)** | 1.013(0.999,1.028) | 0.984(0.963,1.005) |
| 16 | **1.075(1.020,1.132)** | **1.039(1.022,1.055)** | 1.015(1.000,1.031) | 0.982(0.960,1.004) |
| 17 | **1.090(1.027,1.156)** | **1.041(1.023,1.060)** | 1.017(1.000,1.035) | 0.980(0.954,1.006) |
| 18 | **1.105(1.029,1.188)** | **1.044(1.021,1.067)** | 1.019(0.998,1.041) | 0.978(0.947,1.010) |
| 19 | **1.121(1.027,1.224)** | **1.046(1.018,1.075)** | 1.021(0.995,1.048) | 0.976(0.938,1.016) |
| 20 | **1.138(1.022,1.266)** | **1.049(1.015,1.084)** | 1.024(0.992,1.056) | 0.974(0.928,1.022) |
| 21 | **1.154(1.016,1.310)** | **1.051(1.011,1.093)** | 1.026(0.988,1.065) | 0.971(0.918,1.028) |

Values in bold are statistically significant at the *P* < 0.05 level.

**Table S8.** Single-day Lag14 relative risks of T10, N90 and T10_N90 in sensitivity analysis by different model specifications: main model, 14-day washout, and 28-day washout. [RR(95%CI)]

| **Variables** | **Main model** | **14-day washout** | **28-day washout** |
| --- | --- | --- | --- |
| **T10** | **1.011(1.005,1.018)** | **1.012(1.006,1.018)** | **1.013(1.007,1.020)** |
| **N90** | 1.000(0.993,1.007) | 1.000(0.993,1.007) | 1.000(0.993,1.007) |
| **T10_N90** | **1.017(1.004,1.030)** | **1.016(1.004,1.029)** | **1.018(1.005,1.032)** |

The covariates and the *k* of all covariates are consistent with the main model. 14-day washout: we excluded patient data from repeated outpatient visits within a 14-day washout period, retaining only the first visit for each patient during that interval. 28-day washout: we excluded patient data from repeated outpatient visits within a 28-day washout period, retaining only the first visit for each patient during that interval. Values in bold are statistically significant at the *P* < 0.05 level.

**Table S9.** Single-day Lag14 relative risks of exposure to T10, N90 and T10_N90 on the outpatient visits for psoriasis in sensitivity analysis by changing air pollutants. [RR(95%CI)]

| **Variables** | **Main model** | **No pollutant adjustment** | **PM_10_ replace PM_2.5_** | **Add SO_2_** | **Add O_3_** | **Add SO_2_ and O_3_** |
| --- | --- | --- | --- | --- | --- | --- |
| **T10** | **1.011(1.005,1.018)** | **1.014(1.008,1.020)** | **1.011(1.005,1.017)** | **1.011(1.005,1.017)** | **1.009(1.003,1.015)** | **1.009(1.003,1.015)** |
| **N90** | 1.000(0.993,1.007) | 0.997(0.990,1.004) | 1.001(0.994,1.008) | 0.999(0.992,1.006) | 1.002(0.995,1.009) | 1.001(0.994,1.008) |
| **T10_N90** | **1.017(1.004,1.030)** | **1.017(1.004,1.030)** | **1.016(1.004,1.029)** | **1.017(1.004,1.030)** | **1.016(1.004,1.029)** | **1.015(1.003,1.028)** |

PM_10_, particulate matter with an aerodynamic diameter of 10 μm or less; PM_2.5_, particulate matter with an aerodynamic diameter of 2.5 μm or less; SO_2_, sulfur dioxide; O3, ozone. No pollutant adjustment means remove all pollutant covariates; after adjusting for pollutants in the covariates, the other covariates and the *k* of all covariates are consistent with the main model. Values in bold are statistically significant at the *P* < 0.05 level.

**Table S10.** Single-day Lag14 relative risks of T10, N90 and T10_N90 on the outpatient visits for psoriasis in sensitivity analysis by changing the *k* of time. [RR(95%CI)]

| **Variables** | ***k*=4** | ***k* =5 (main model)** | ***k*=6** | ***k*=7** |
| --- | --- | --- | --- | --- |
| **T10** | **1.011(1.005,1.017)** | **1.011(1.005,1.018)** | **1.011(1.005,1.017)** | **1.012(1.006,1.017)** |
| **N90** | 1.000(0.993,1.007) | 1.000(0.993,1.007) | 1.000(0.993,1.007) | 1.000(0.993,1.007) |
| **T10_N90** | **1.017(1.004,1.030)** | **1.017(1.004,1.030)** | **1.017(1.004,1.030)** | **1.018(1.006,1.031)** |

When the *k* of the time was adjusted, the *k* for relative humidity, PM_2.5_ and CO are consistent with the main model. Values in bold are statistically significant at the *P* < 0.05 level.

**Table S11.** Single-day odds ratios [OR(95%CI)] and the global *P* value of T10_N90 on the high systemic inflammation of psoriasis in the outpatient visits.

| **Lag** | **High NLR** | **High PLR** | **High SII** |
| --- | --- | --- | --- |
| 0 | 1.065(0.890,1.274) | 1.043(0.879,1.238) | 1.165(0.979,1.386) |
| 1 | 1.062(0.913,1.236) | 1.042(0.901,1.203) | 1.157(0.999,1.341) |
| 2 | 1.060(0.934,1.202) | 1.040(0.922,1.173) | **1.150(1.017,1.299)** |
| 3 | 1.057(0.953,1.173) | 1.038(0.940,1.146) | **1.142(1.033,1.263)** |
| 4 | 1.055(0.967,1.150) | 1.036(0.954,1.126) | **1.134(1.043,1.233)** |
| 5 | 1.052(0.975,1.136) | 1.034(0.961,1.113) | **1.126(1.046,1.213)** |
| 6 | 1.050(0.976,1.129) | 1.032(0.962,1.108) | **1.118(1.042,1.200)** |
| 7 | 1.048(0.972,1.130) | 1.030(0.958,1.108) | **1.109(1.031,1.194)** |
| 8 | 1.046(0.966,1.133) | 1.028(0.951,1.111) | **1.100(1.018,1.190)** |
| 9 | 1.045(0.961,1.137) | 1.026(0.945,1.114) | **1.091(1.004,1.185)** |
| 10 | 1.044(0.957,1.138) | 1.023(0.940,1.114) | 1.081(0.993,1.177) |
| 11 | 1.043(0.957,1.137) | 1.020(0.938,1.110) | 1.071(0.984,1.165) |
| 12 | 1.043(0.960,1.132) | 1.018(0.939,1.103) | 1.060(0.977,1.149) |
| 13 | 1.043(0.966,1.126) | 1.015(0.942,1.094) | 1.048(0.972,1.131) |
| 14 | 1.043(0.971,1.120) | 1.012(0.944,1.084) | 1.037(0.966,1.113) |
| 15 | 1.043(0.974,1.118) | 1.008(0.942,1.079) | 1.025(0.956,1.098) |
| 16 | 1.044(0.970,1.123) | 1.005(0.935,1.080) | 1.013(0.940,1.091) |
| 17 | 1.045(0.960,1.138) | 1.002(0.921,1.090) | 1.000(0.916,1.092) |
| 18 | 1.046(0.942,1.160) | 0.998(0.900,1.107) | 0.988(0.887,1.099) |
| 19 | 1.047(0.921,1.189) | 0.995(0.876,1.129) | 0.975(0.855,1.112) |
| 20 | 1.048(0.898,1.223) | 0.991(0.850,1.155) | 0.963(0.822,1.128) |
| 21 | 1.049(0.873,1.260) | 0.988(0.824,1.184) | 0.951(0.788,1.147) |
| ^b^*P* | 0.331 | 0.834 | **0.016** |

NLR, neutrophil-to-lymphocyte ratio; PLR, platelet-to-lymphocyte ratio; SII, systemic immune-inflammation index. ^b^*P*, Global *P*‑values were derived from the quasi‑likelihood F‑test for the cross‑basis term, testing the joint significance of the exposure across all 21 lag days. Values in bold are statistically significant at the *P* < 0.05 level.
